# Deciphering *Bartonella* Diversity, Recombination, and Host Specificity in a Rodent Community

**DOI:** 10.1371/journal.pone.0068956

**Published:** 2013-07-24

**Authors:** Jean-Philippe Buffet, Benoît Pisanu, Sylvain Brisse, Sophie Roussel, Benjamin Félix, Lénaïg Halos, Jean-Louis Chapuis, Muriel Vayssier-Taussat

**Affiliations:** 1 USC INRA Bipar, Bartonella et Tiques, Maisons-Alfort, France; 2 UMR 7204 MNHN-CNRS-P6, Conservation des Espèces, Restauration et Suivi des Populations, Muséum National d'Histoire Naturelle, Paris, France; 3 Genotyping of Pathogens and Public Health, Institut Pasteur, Paris, France; 4 Laboratory for Food Safety, Agence Nationale de Sécurité Sanitaire de l'Alimentation, de l'Environnement et du Travail (ANSES), Maisons-Alfort, France; 5 MERIAL, Lyon, France; University of Helsinki, Finland

## Abstract

Host-specificity is an intrinsic feature of many bacterial pathogens, resulting from a long history of co-adaptation between bacteria and their hosts. Alpha-proteobacteria belonging to the genus *Bartonella* infect the erythrocytes of a wide range of mammal orders, including rodents. In this study, we performed genetic analysis of *Bartonella* colonizing a rodent community dominated by bank voles (*Myodes glareolus*) and wood mice (*Apodemus sylvaticus*) in a French suburban forest to evaluate their diversity, their capacity to recombine and their level of host specificity. Following the analysis of 550 rodents, we detected 63 distinct genotypes related to *B. taylorii*, *B. grahamii*, *B. doshiae* and a new *B. rochalimae*-like species. Investigating the most highly represented species, we showed that *B. taylorii* strain diversity was markedly higher than that of *B. grahamii,* suggesting a possible severe bottleneck for the latter species. The majority of recovered genotypes presented a strong association with either bank voles or wood mice, with the exception of three *B. taylorii* genotypes which had a broader host range. Despite the physical barriers created by host specificity, we observed lateral gene transfer between *Bartonella* genotypes associated with wood mice and *Bartonella* adapted to bank voles, suggesting that those genotypes might co-habit during their life cycle.

## Introduction

Bacterial strain diversification within their natural host populations can restrict the potential future range of hosts that are susceptible to infection. As a result of adaptive evolution in their principal host, bacteria have a typically limited host range that they can successfully infect. At the molecular level, such host-parasite adaptation is reflected by specific receptor-ligand interactions between bacterial factors and their target host factors [1]. The resulting host specificity is an inherent feature of many bacterial pathogens, including species of the genus *Bartonella*. Although largely unexplored, the *Bartonella* species represents an interesting model for investigating the patterns and processes of host specificity [2].

*Bartonella* species infect mammalian reservoirs, including rodents, in which they induce an asymptomatic long lasting intra-erythrocytic bacteremia [3]. To date, more than 30 species and subspecies of *Bartonella* have been partially or completely characterized. Thirteen of which are recognized as emerging zoonotic pathogens, causing life-threatening infections in both animal and human populations. *Bartonella* species are mainly transmitted to their mammalian hosts through feces of ectoparasites after superficial scratching of their skin (or occasionally directly by the bite of blood feeding arthropods). They then colonize a primary niche from where they are seeded into the bloodstream before finally adhering to and invading erythrocytes [3], [4]. Infection is driven by two main pathogenic factors belonging to the type IV secretion systems (T4SS): the VirB/VirD4, involved in primary niche invasion and adaptation to the mammalian host, and the Trw T4SS, involved in host-specific erythrocyte invasion.

As a result of hypothesized adaptive radiation [5], each of the 30 *Bartonella* species or subspecies only infects one, or a few closely related mammalian host reservoirs [3], [6]. We have recently demonstrated that bartonellae host specificity is driven by their unique ability to adhere and infect erythrocytes from their natural host(s) via the Trw T4SS [7]. Equally important for *Bartonella* species host adaptation is the VirB T4SS machinery, which translocates a cocktail of related effector proteins into primary niche host cells, where they modulate various processes, resulting in the ability to adapt to a wide range of different hosts [5], [8].

While some species are highly specific for their host (i.e., *B. henselae* for the cat or *B. bacilliformis* for humans), other specific associations do not seem so clear-cut, especially for rodent-associated species. In addition the association between some *Bartonella* species and their rodent hosts remains a matter of controversy. Indeed, while *B. vinsonii* subsp. *arupensis* and *B. washoensis* have only been found to infect *Peromyscus* mice and ground squirrels respectively [9]–[12], *B. elizabethae* has been found to infect both *Rattus* spp. rats in Southern China [13] and *Bandicota* spp. rats in Thailand. In Europe, *B. taylorii*, *B. grahamii* and *B. doshiae* infect several sympatric woodland rodents at a single site [14]–[17], while a longitudinal study realized in Poland reported a strong host association between the majority of *Bartonella* species and their rodents (*Apodemus* mice and *Myodes* voles) [18]. Recently, the complete genome sequences of two rodent-carried *Bartonella* species (i.e. *B. tribocorum* and *B. grahamii*) were compared to *Bartonella* species from humans and cats, revealing that the rodent-associated species carried a higher number of T4SS host-adaptability factor encoding genes [19]. Interestingly, these host-adaptability genes are packaged into bacteriophage particles, resulting in an original method of gene exchange between *B. grahamii* strains promoting rapid diversification. This could therefore facilitate host shifting, which may explain the observed lack of host specificity of some species or strains [19].

In order to improve our understanding of how *Bartonella* species are associated with their mammalian hosts, it is important to accurately describe *Bartonella* species diversity within their natural host. Previous multi-locus sequence typing methods (MLST) of *B. henselae* strains isolated from humans and cats sampled across several continents revealed 14 genotypes, but did not disclose an obvious host association pattern, except for one genotype restricted to European cats [20]. However, a more recent MLST scheme based on eight loci indicated that the majority of human isolates are rare genotypes differing from those of cats [21]. Moreover, a variable number tandem repeat analysis performed on 178 *B. henselae* strains resulted in 99 profiles separated into two groups, where all human isolates were clustered within the same group [22]. All typing schemes applied to *B. quintana* strains, strictly human specific, indicated very low levels of diversity [23], [24]. In relation to rodent-adapted *Bartonella* species, the only published study utilized multi-locus sequence analysis (MLSA) of *B. grahamii* strains sampled worldwide, and demonstrated strict host specificity in Asia and North America, and low host specificity in Europe [25], [26].

Typically, intra-cellular bacteria have lower recombination rates, probably due to their relative niche isolation [27] and indeed, the *Bartonella* genus has one of the lowest recombination rates among bacteria [27]. This might be due to its intracellular location but also to its restricted host range. Interestingly, within the *Bartonella* genus, strains circulating within rodents showed more frequent recombination events compared to human and cat adapted species, suggesting a broader host range for rodent adapted species [26], [28].

To learn more about the adaptation of *Bartonella* species to their wild animal reservoirs, we conducted a field study in which *Bartonella* genotypes were identified from a rodent community living within a suburban forest. To determine the level of genotype specificity for rodent species, we performed robust genetic diversity analysis on the recovered *Bartonella* strains, based on a Multi-locus Sequence Analysis (MLSA) using six housekeeping genes. Recombination events between strains were analyzed to evaluate whether these strains have had the opportunity to co-habit. Finally, due to the important role of the T4SS VirB/D4 in *Bartonella* species host adaptability, we also investigated whether the pilus component VirB5 diversity revealed a possible host association.

## Materials and Methods

### Study site and animal processing

The study was conducted in 2007 and 2008 in the Forest of Sénart (3,200 ha, 02°29′E, 48°40′N), located in a dense urbanized area 22 km south-east of Paris [17]. Small mammals were collected from 5 sites in communities dominated by oaks (*Quercus petraea, Q. robur*) associated with hornbeam (*Carpinus betulus*) (sites 1, 3 and 5), or associated with the chestnut (*Castanea sativa*) (sites 2 and 4). The trappings were conducted in April 2008 at sites 1 to 4 and from March to October 2007 and 2008 on the site 5 [29], [30].

A set of small rodents were sacrificed by cervical dislocation: 68 bank voles (*Myodes glareolus*) and 70 wood mice (*Apodemus sylvaticus*) in April 2008 on sites 1–4, and 402 bank voles and 9 field voles (*Microtus agrestis*) on site 5 between 2007 and 2008 (Table 1). No wood mouse, strictly nocturnal, has been collected on the site 5 where traps were closed during the night, because the target species of this field design was the Siberian chipmunk (*Tamias sibiricus*) which is diurnal. Abundance of wood mice and bank voles were similar at sites 1 to 4 in April 2008. Sacrificed rodents were frozen at −20°C before analysis. In the laboratory, the spleen was removed under sterile conditions, and stored at −80°C until they were to be used for *Bartonella* culture and/or DNA PCR detection.

**Table 1:** Distribution of species and clusters of *Bartonella* in the rodent community in the Forest of Sénart.

Species	n	*taylorii*					*doshiae*	*grahamii*	*rochalimae*-like
Clusters		*A*	*B*	*C*	*D*	*E*			
**Site 1–4 (April 2008)**									
*A. sylvaticus *(wood mice)	70	3	0	0	0	6	0	0	0
*M. glareolus *(bank voles)	69	12	4	8	8	0	2	1	1
**Site 5 (2007–2008)**									
*M. glareolus *(bank voles)	402	38	14	23	34	0	4	28	0
*Mi. Agrestis* (field voles)	9	0	0	0	1	0	0	0	0

### Bacterial isolates and culture conditions

Bacterial isolation was performed by grinding a piece of spleen in 500 µl of F-12 nutrient mixture medium (Invitrogen), and then plating 250 µl of the mixture onto Colombia agar containing 5% defibrinated sheep's blood (CBA), which was then incubated at 35°C in a humidified atmosphere with 5% CO_2_ up to 30 days (plates being checked every day from day 5).

### PCR amplification and sequencing of protein-coding genes

DNA was extracted from rodent spleen samples as previously described [17], and bacterial isolates was heated in 200 µl suspension at 95°C for 10 min, followed by a 5 min centrifugation step of 6,000 *g* at 4°C. The supernatant was then collected and used for PCR. The presence of *Bartonella* DNA was determined by using a portion of the *gltA* gene as previously described [17]. For all positive samples, five other protein-coding gene sequences were amplified for MLSA analysis: cell division protein gene (*ftsZ*), NADH dehydrogenase gamma-subunit gene (*nuoG*), 60 kDa heat-shock protein gene (*groEL*), riboflavin synthase gene (*ribC*), and RNA polymerase beta-subunit gene (*rpoB*) using primers previously described [28], [31]–[34].

For *virB5* analysis, the 447–522 bp region was amplified and sequenced from 63 DNA samples (or isolate when avalaible) representing the diversity of *Bartonella* strains recovered in this study. To amplify the complete *virB5* gene, primers were designed as follows: the forward primer virB5F-B4: 5′-GCA-GAA-CTY-AAY-TTA-CGK-GG-3′ was complementary to the 3′ end of the *virB4* gene (accession numbers, NC_012846, NC_010161, NC_014932, NC_005956), and the reverse primer virB5R3-B6: 5′-GCA-TTY-GTT-GCC-ATT-GTT-GTC-AC-3′ complemented the 5′ end of the *virB6* gene (accession numbers, NC_012846, NC_010161, NC_014932, NC_005956), resulting in complete coverage of the *virB5* gene.

PCR amplification was performed in a final volume of 25 µl containing 0.5 µM of each primer, 3% (vol/vol) of dimethyl sulfoxide, 200 µM of each deoxynucleotide triphosphate, 1x Phusion^TM^ GC buffer, 0.4 U of Phusion^TM^ DNA polymerase (Finnzymes) and 20–50 ng of genomic DNA. The PCR amplification conditions were as follows: an initial cycle of 95°C for 5 min; 37 amplification cycles, each consisting of 95°C for 30 s, 54°C (*gltA*), 55°C (*ftsZ*), 54°C (*groEL*), 53°C (*rpoB*), 48°C (*ribC*), 53°C (*nuoG*) and 48°C (*virB5*) for 30 s, followed by an elongation step of 72°C for 1 min; and a final incubation at 72°C for 10 min. Amplification products were analyzed by electrophoresis on 2.5% agarose gels with 0.1 mg/ml of ethidium bromide and verified under UV light. PCR products were then purified and sequenced on both strands by Eurofins MWG Operon (Germany).

To validate all amplified sequences, we repeated DNA extraction, amplification and sequencing for each spleen sample and isolate.

### Phylogenetic analysis and recombination tests

Sequences were edited and aligned using Geneious^TM^ v5.6.4 software. Nucleotide diversity indices and the polymorphic level were calculated using DNAsp v5.10 [35]. Neighbor-joining tree analysis for each individual gene was performed using MEGA v5 [36]. For the MLSA approach, individual gene sequences were concatenated. The best model of nucleotide substitution was determined with jModelTest [37]. A phylogeny was constructed based on the concatenates by using the maximum-likelihood method implemented in PhyML software [38]. Missing genes were considered as deletions. *Bartonella* reference strain sequences are listed in Table S1 in File S1.

To build the phylogeny of *virB5* sequences, the best model of nucleotide substitution was calculated with jModelTest, and the phylogeny constructed using the maximum-likelihood method with MEGA v5.

A phylogenetic tree was also inferred using ClonalFrame v1.1 [39] with the number of MCMC iterations set to 200,000, following a burn-in period of 100,000 iterations. ClonalFrame is a Bayesian inference method which jointly reconstructs the clonal relationships between the isolates in a sample, as well as the location of recombination events that have disrupted the clonal signal.

Allelic profiles were identified for each individual *Bartonella* strain, and an allele number was assigned to every distinct sequence variant for each of the six loci, using the BioNumerics v6.5 software (Applied-Maths, Sint Maartens-Latem, Belgium). To test for linkage disequilibrium between alleles of the six analyzed loci, the Index of association (Ia) between alleles [40] was calculated using the START program (http://pubmlst.org).

Recombination test calculations were performed using RDP3 [41]. The Neighbor-Net implemented in the software SplitsTree 4.12 [42] with 1,000 bootstrap replicates was used to create the phylogenic network for the concatenated sequences. Furthermore, we used the pairwise homoplasy index (PHI) [43] in SplitsTree 4.0 in order to test the role of past recombination events in generating allelic variation. To estimate the relative contribution of recombination and mutation events among *Bartonella* genotypes, we performed two independent runs of the analysis tool ClonalFrame [39], each consisting of 200,000 MCMC iterations, discarding the first half as burn-in. We used the linkage model in Structure v2.3 [44] to identify populations with distinct allele frequencies present in our data and estimate genetic exchange among these populations. We performed five runs, each using a different value of between two and eight for the number of populations (*K*), each with 100,000 MCMC iterations, following a burn-in period of 50,000 iterations. These tests indicated that the model probability was highest at *K* = 4.

### Nucleotide sequences

Sequences of the six protein-coding genes and the *virB5* gene were deposited in GenBank/EMBL/DDBJ databases under the accession numbers JX846090 to JX846493 (Table S2 in File S1).

## Results

Among the 550 analyzed rodents, 195 DNA extracts were amplified using *Bartonella-gltA* specific primers (i.e., 35.5% of the rodent community). Considering each rodent species, prevalence was 11% for field voles (1/9); 39,3% for bank voles (185/471) and 12,8% for wood mice (9/70). Bacterial isolation was possible from 43 out of the 195 *gltA*-positive spleen samples.

### Phylogenetic relationships of *Bartonella* genotypes, delineation of genetic clusters

**Figure 1:**
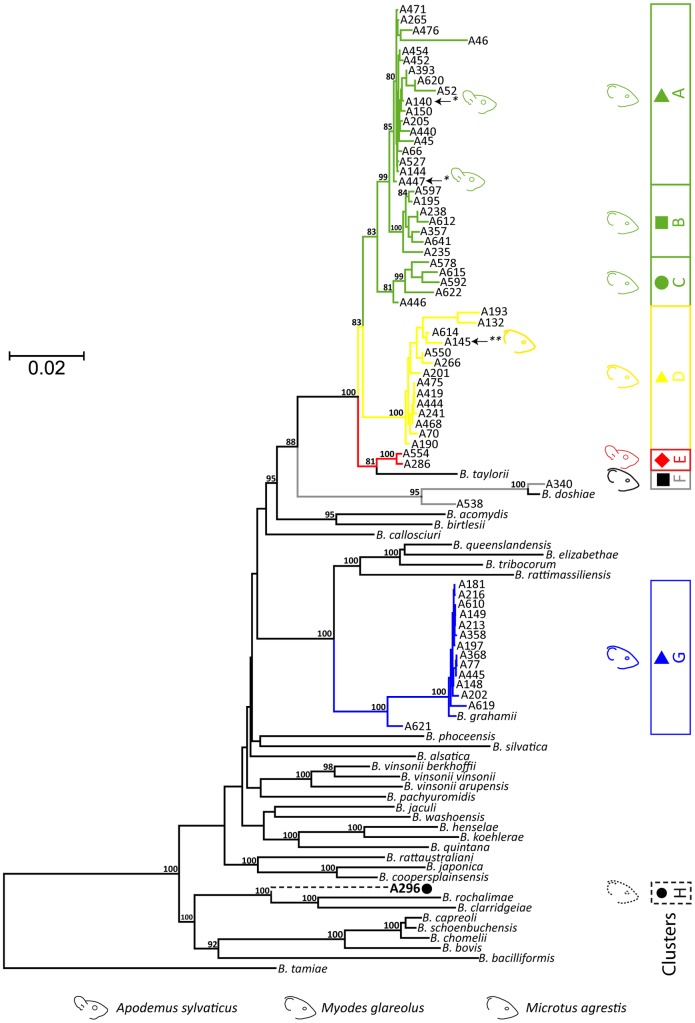
Phylogenetic analysis of representative genotypes circulating in the rodent community. Maximum-likelihood analysis of 63 *Bartonella* unique genotypes detected in this study and 19 *Bartonella* species using the alignment of concatenated sequences of six loci (*ftsZ*, *gltA*, *groEL*, *nuoG*, *ribC* and *rpoB*). The numbers at the nodes correspond to bootstrap values higher than 80%.

To characterize the diversity of *Bartonella* circulating in the rodent community, we first performed a phylogenetic analysis based on the alignment of the concatenated sequences of the six housekeeping genes, using the 195 *Bartonella gltA*-positive DNA extracts and the corresponding *Bartonella* strains when available. The phylogenetic tree (Figure 1) was generated using maximum likelihood (PhyML) with a K81 substitution model (estimated using jModelTest). Only one representative of each genotype was reported on the phylogenetic tree. This analysis revealed the phylogenetic relationships of the 63 unique genotypes with three known *Bartonella* species: *B. taylorii*, *B. doshiae*, and *B. grahamii*; and a genotype (A296, cluster H) closely related to *B. rochalimae* and *B. clarridgeiae* (8.2 and 9.3% nucleotide divergence respectively). *B. grahamii* (cluster G) did not exhibit an obvious internal phylogenetic structure, except for one genotype (A621) recovered from a single bank vole, which was genetically diverged from all the other *B. grahamii* genotypes by 2.4 to 2.9%. In contrast, within the *B. taylorii* species, the phylogenetic tree showed a clear demarcation of five clades (A, B, C, D and E) with high bootstrap support (81–100%). Each clade formed distinct compact clusters separated by genetic distances ranging from 1.3 to 3.5%. As a MLSA-based genetic distance below 5% is sufficient to join a genotype to a known species, all these clusters clearly belong to *B. taylorii* species [45]. Two genotypes were associated with the species *B. doshiae* (cluster F), of which one (A340) was more closely related to the R18 reference strain with a genetic distance of 0.7%, and another (A538) more distant which diverged by a distance of 4.5%. Distribution of *Bartonella* species and clusters in the rodent community is summarized in Table 1. The 63 different genotypes, the rodent species from where they were detected or isolated (as well the number of animal in which they were recoververd) and the corresponding *Bartonella* species and clusters are descripted in Table 2.

**Table 2:** Description of the 63 genotypes of *Bartonella* identified in rodents.

Related genotypes	Strain isolation	Rodent species	*Bartonella* sp. (cluster)	Number of infected animals
A132	Yes	*M. glareolus*	*B. taylorii* (D)	2
A140		*M. glareolus/A. sylvaticus*	*B. taylorii* (A)	2
A144		*M. glareolus*	*B. taylorii* (A)	1
A145		*M. glareolus/M. agrestis*	*B. taylorii* (D)	7
A148	Yes	*M. glareolus*	*B. grahamii* (G)	1
A149		*M. glareolus*	*B. grahamii* (G)	3
A150		*M. glareolus*	*B. taylorii* (A)	1
A181	Yes	*M. glareolus*	*B. grahamii* (G)	2
A190		*M. glareolus*	*B. taylorii* (D)	1
A193	Yes	*M. glareolus*	*B. taylorii* (D)	1
A195		*M. glareolus*	*B. taylorii* (B)	1
A197	Yes	*M. glareolus*	*B. grahamii* (G)	1
A201		*M. glareolus*	*B. taylorii*(D)	1
A202	Yes	*M. glareolus*	*B. grahamii* (G)	2
A205		*M. glareolus*	*B. taylorii* (A)	1
A213	Yes	*M. glareolus*	*B. grahamii* (G)	4
A216	Yes	*M. glareolus*	*B. grahamii* (G)	2
A235		*M. glareolus*	*B. taylorii* (B)	1
A238		*M. glareolus*	*B. taylorii* (B)	1
A241		*M. glareolus*	*B. taylorii* (D)	1
A265		*M. glareolus*	*B. taylorii* (A)	1
A266		*M. glareolus*	*B. taylorii* (D)	7
A286	Yes	*A. sylvaticus*	*B. taylorii* (E)	6
A296	Yes	*M. glareolus*	*B. rochalimae*-like (H)	1
A340	Yes	*M. glareolus*	*B. doshiae* (F)	2
A357	Yes	*M. glareolus*	*B. taylorii* (B)	13
A358		*M. glareolus*	*B. grahamii* (G)	5
A368	Yes	*M. glareolus*	*B. grahamii* (G)	1
A393	Yes	*M. glareolus*	*B. taylorii* (A)	30
A419	Yes	*M. glareolus*	*B. taylorii* (D)	1
A440		*M. glareolus*	*B. taylorii* (A)	1
A444		*M. glareolus*	*B. taylorii* (D)	1
A445	Yes	*M. glareolus*	*B. grahamii* (G)	2
A446	Yes	*M. glareolus*	*B. taylorii* (C)	8
A447	Yes	*M. glareolus/A. sylvaticus*	*B. taylorii* (A)	13
A45		*M. glareolus*	*B. taylorii* (A)	1
A452		*M. glareolus*	*B. taylorii* (A)	1
A454		*M. glareolus*	*B. taylorii* (A)	1
A46		*M. glareolus*	*B. taylorii* (A)	1
A468		*M. glareolus*	*B. taylorii* (D)	3
A471		*M. glareolus*	*B. taylorii* (A)	1
A475		*M. glareolus*	*B. taylorii* (D)	1
A476		*M. glareolus*	*B. taylorii* (A)	1
A52	Yes	*M. glareolus*	*B. taylorii* (A)	1
A527		*M. glareolus*	*B. taylorii* (A)	1
A538	Yes	*M. glareolus*	*B. doshiae* (F)	4
A550	Yes	*M. glareolus*	*B. taylorii* (D)	6
A554	Yes	*A. sylvaticus*	*B. taylorii* (E)	3
A578		*M. glareolus*	*B. taylorii* (C)	1
A592		*M. glareolus*	*B. taylorii* (C)	11
A597		*M. glareolus*	*B. taylorii* (B)	1
A610	Yes	*M. glareolus*	*B. grahamii* (G)	1
A612		*M. glareolus*	*B. taylorii* (B)	1
A614		*M. glareolus*	*B. taylorii* (D)	8
A615		*M. glareolus*	*B. taylorii* (C)	3
A619		*M. glareolus*	*B. grahamii* (G)	2
A620		*M. glareolus*	*B. taylorii* (A)	1
A621		*M. glareolus*	*B. grahamii* (G)	2
A622		*M. glareolus*	*B. taylorii* (C)	8
A641		*M. glareolus*	*B. taylorii* (B)	1
A66		*M. glareolus*	*B. taylorii* (A)	1
A70		*M. glareolus*	*B. taylorii* (D)	1
A77	Yes	*M. glareolus*	*B. grahamii* (G)	1

The description includes the rodent species and the number of animals from where gentoypes were detected or isolated, as well as the *Bartonella* species and clusters of the corresponding genotype.

### Host specificity of *Bartonella* genotypes

When considering the specific association between *Bartonella* species and their rodent hosts, all *B. grahamii*, *B. doshiae* and the *B. rochalimae* related species genotypes were only recovered from bank voles. At the species level, *B. taylorii* strains were recovered from field voles, bank voles and wood mice with an apparent host preference for bank voles (93.3 % of *B. taylorii* strains were isolated from bank voles). However, data seem to indicate host specificity at the genotype level with *B. taylorii* clusters B and C only recovered from bank voles, while cluster E has been only isolated from wood mice. Clusters A and D have a larger host range and are detected in bank voles, wood mice (cluster A) or field voles (cluster D) (Figure 1, Table 2). Interestingly, genotypes infecting wood mice (A140 and A447) from cluster A and genotypes infecting field voles (A145) from cluster D can also infect bank voles (Figure 1, Table 2).

### Genetic polymorphism and recombination within rodent adapted *Bartonella* species

We examined sequence polymorphism of the six housekeeping genes in all genotypes corresponding to *B. taylorii* and *B. grahamii*. *B. doshiae* and *B. rochalimae* related species were excluded from the study because of the low number of genotypes. Following alignment of the 3448 nucleotides of the six gene fragments, a total of 793 (23%) polymorphic sites were found, discriminating 41 individual alleles (Table 3). The number of polymorphic sites on a given locus varied from 56 (17.2%) for *nuoG* to 217 (26.72%) for *groEL*. The number of individual alleles for each of the six protein-coding genes ranged from 8 for *ftsZ* to 28 for *groEL*. Most polymorphisms resulted in synonymous substitutions, and the ratio of non-synonymous to synonymous substitutions (*dN*/*dS*) varied from 0.016 (for *nuoG*) to 0.24 (for *rib*C) (Table 3). These low ratios indicate a combination of purifying selection on amino-acid variation and a lack, or a very limited contribution, of positive environmental selection to the sequence variation in the six analyzed loci. These genes are therefore assumed to be appropriate for a population genetic study.

**Table 3:** Polymorphism and nucleotide diversity of six housekeeping protein-coding genes among *Bartonella* genotypes.

Genes	Size	N.	Polymorphic (non synonymous) sites		dN	dS	dN/dS	π (%)
	(bp)	alleles	N	%				
**All genotypes (n = 64)**								
*ftsZ*	788	8	147	18.65	0.0064	0.1623	0.0396	4.405
*gltA*	326	11	73	22.39	0.0128	0.2064	0.0619	5.419
*groEL*	812	28	217	26.72	0.0118	0.1520	0.0775	4.625
*nuoG*	325	16	56	17.23	0.0037	0.2262	0.0164	5.801
*ribC*	408	18	138	33.82	0.0428	0.1816	0.2358	7.562
*rpoB*	788	9	162	20.56	0.0080	0.1785	0.0449	4.952
Concatenate	3448	41	793	23.00	0.0127	0.1758	0.0724	5.182
***B. taylorii*** ** (n = 48)**								
*ftsZ*	788	4	26	3.30	0.0001	0.0376	0.0037	0.920
*gltA*	326	7	32	9.82	0.0065	0.0790	0.0820	2.202
*groEL*	812	22	112	13.79	0.0101	0.0175	0.5784	1.199
*nuoG*	325	14	51	15.69	0.0047	0.1583	0.0296	4.208
*ribC*	408	13	98	24.02	0.0158	0.0597	0.2652	2.618
*rpoB*	788	6	90	11.42	0.0020	0.0570	0.0351	1.542
Concatenate	3448	33	409	11.86	0.0058	0.0548	0.1062	1.760
***B. grahamii*** ** (n = 16)**								
*ftsZ*	788	2	1	0.13	0.0002	0.0000	NA	0.016
*gltA*	326	2	2	0.61	0.0005	0.0018	0.2722	0.077
*groEL*	812	4	9	1.11	0.0002	0.0114	0.0175	0.296
*nuoG*	325	1	0	0.00	0.0000	0.0000	NA	0.000
*ribC*	408	3	17	4.17	0.0050	0.0070	0.7133	0.547
*rpoB*	788	2	1	0.13	0.0000	0.0024	0.0000	0.058
Concatenate	3448	7	30	0.87	0.0007	0.0043	0.1733	0.159

π, average number of nucleotide differences per site; dS, number of synonymous changes per synonymous site; dN, number of non-synonymous changes per non-synonymous site; NA, not applicable.

The average nucleotide diversity (π) within all *Bartonella* genotypes was 5.2%±0.471, ranging from 4.4% to 7.5% per gene. Within the species *B. grahamii*, the average nucleotide distance was restricted (π = 0.159%±0.054, Table 3), indicating that the core genome of *B. grahamii* is relatively homogeneous, whereas *B. taylorii* (π = 1.76%±0.211) was diverse. We also noted striking intergene variation in levels of diversity within *B. taylorii* and *B. grahamii* genotypes: *nuoG* (π = 4.2%±0.458) sequences were found to be more variable than sequences of *gltA* (2.2%±0.255 on average) within *B. taylorii* species; within *B. grahamii*, *groEL* sequences (0.3±0.077) were more heterogeneous than those of *gltA* (0.077%±0.065), whereas *nuoG* sequences (1 allele, no polymorphic site) were homogeneous.

A quantitative analysis of the association between alleles from the six loci, by calculating the Index of Association (Ia) [40], estimated the contribution of recombination to the genetic structure among individuals of a population. Including all the *Bartonella* strains in the analysis, significant linkage disequilibrium was detected (Ia  = 1.999±0.846), as expected for distinct species. Within *B. taylorii* genotypes, Ia was calculated as 1.847±0.988, confirming the significant linkage disequilibrium between alleles and hence a deviation from a random-mixing population within this species. In contrast, among *B. grahamii* strains, with a value of Ia at 0.128±0.761, no significant linkage disequilibrium was detected indicating frequent homologous recombination events.

### Relationships between host specificity of rodent adapted *Bartonella* species and homologous recombination among clusters

**Figure 2:**
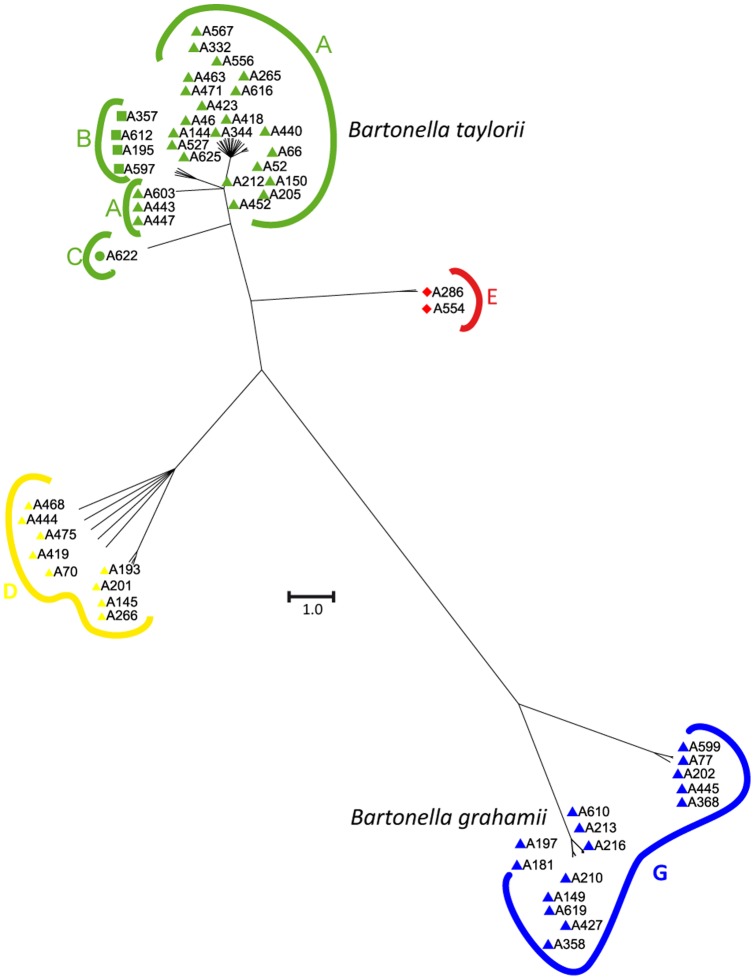
50% consensus tree from ClonalFrame.

Throughout the entire *Bartonella* genotypes, the inferred recombination to mutation value (*r*/*m* ratio) calculated with ClonalFrame was estimated at 4.06 (CI_95_  =  [2.28; 6.38]), suggesting that nucleotide changes in housekeeping genes occur more frequently by recombination than by *de novo* mutation. Among the rodent community's two most abundant *Bartonella* species, recombination rather than mutation was found to have played a more important role within *B. taylorii* as compared to *B. grahamii* (*r*/*m*  = 6.81 and 3.77, respectively). To examine any possible influences of recombination on the tree topology (Figure 1), we inferred a phylogenetic tree of the complete dataset using ClonalFrame, which takes recombination into account during tree building. Analysis resulted in a tree with the same major clades as identified by MLSA (Figure 2). We then evaluated whether recombination was detected between clusters by using several different approaches. Firstly, we individually compared the phylogeny of each gene and demonstrated that *Bartonella* genotype position varied depending upon the gene (Figure S1). Indeed, individual *ftsZ*, *gltA*, *ribC* and *rpoB* phylogenies showed strong congruence among clusters, but with some remarkable discrepancies. For example, as opposed to the concatenate or to five other genes, *gltA* did not strongly associate all of the genotypes belonging to cluster A. In contrast for this phylogeny, genotypes of cluster A were separated into two clades, of which one merged in a short branch with cluster B and C genotypes (Figure S1). This observation could be attributed to the horizontal transfer of the *gltA* gene from a donor related to cluster A, into an ancestral strain of clusters B and C. The *rpoB* sequence of the genotype A538 (*B. doshiae*) was identical to cluster A of *B. taylorii* sequences. Likewise, in *ribC* phylogeny, we identified a different position for 10 genotypes among clusters A, C, D (*B. taylorii*), G (*B. grahamii*) and H (*B. rochalimae*-like). For example, the *ribC* sequences of genotypes A296 (cluster H), A132 and A193 (cluster D) were almost identical to *B. grahamii* sequences, whereas the *ribC* sequence of A621 (*B. grahamii*) was identical to that of *B. taylorii* cluster A genotypes (Figure S1). *GroEL* and *nuoG* phylogenies were more discordant in their phylogenetic placement of genotypes. For the *nuoG* phylogeny, cluster C genotypes were distributed amongst all clusters, and genotype A296 was associated with cluster D. The *groEL* phylogeny showed an association of *B. taylorii* and *B. doshiae* genotypes to the *B. birtlesii* reference strain IBS 325, strongly supported by high bootstrap (Figure S1). These results, which were confirmed by re-sequencing of the six genes from new DNA extracts, demonstrated the occurrence of horizontal gene transfer between distantly related *Bartonella* species, as has been previously described [28].

**Figure 3:**
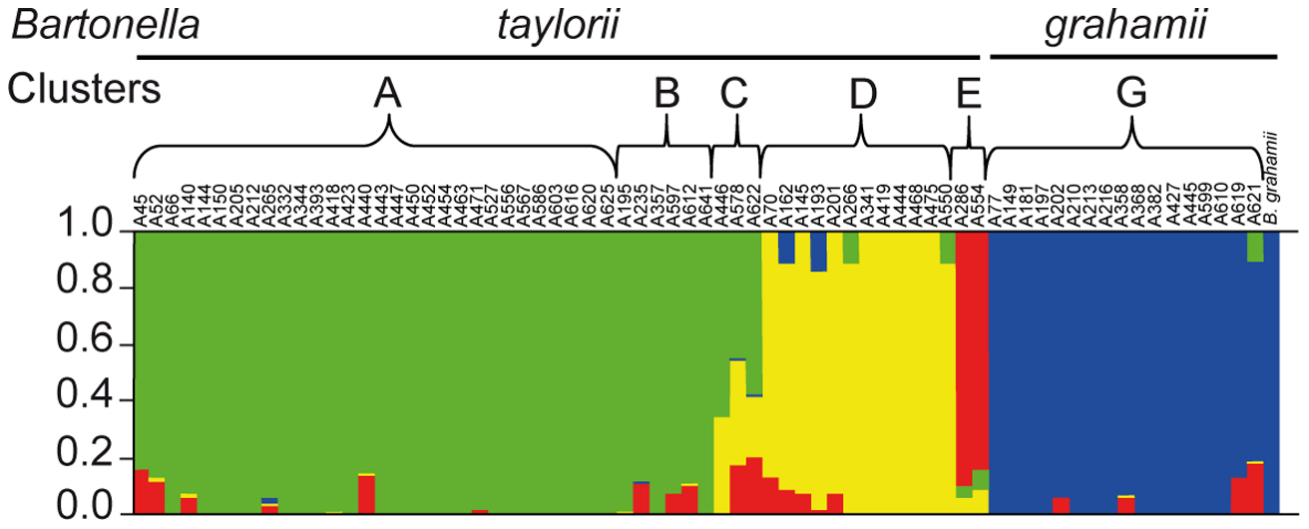
Recombination events between genotypes. Results of Structure analysis showing the inferred proportion of nucleotides from four ancestral populations for each strain. The plot shows one vertical line for each strain, and the length of the colored segments indicates the proportions of nucleotides from each of the four ancestral populations.

Secondly, in order to detect those more subtle recombination events which result in detectable footprints of foreign DNA incorporation, nucleotide polymorphisms within the six genes were analyzed with the Structure software [44]. Structure recognized four clusters (A, D, E and G), and most strains within the identified clusters were homogeneous in terms of their ancestral polymorphism source (Figure 3). However, as expected, the transfer of gene sequences from other clusters were visualized by Structure, as is evident from the mixed color of the corresponding genotype column (Figure 3). Moreover, Structure revealed a number of genotypes that appeared to have a partially mixed origin, such that a mosaic pattern was seen where recombination of a large contiguous part of a gene had occurred. For example, a 323 bp segment of *ribC* from genotype A132 (*B. taylorii*, cluster D, recovered from a bank vole) was nearly identical to *B. grahamii* sequences (cluster G specific to bank voles), while a 293 bp segment of *groEL* was identical to sequences of *B. taylorii* cluster E genotypes (specific to wood mice). Furthermore, genotype A550 (*B. taylorii*, cluster D recovered from bank voles) contained a 313 bp *ribC* segment nearly identical to *B. taylorii* cluster A sequences (recovered from both bank voles and wood mice). Likewise, genotype A621 (*B. grahamii*, cluster G) exhibited a mixed origin, with *ribC* sequences matching those in *B. taylorii* clusters A and E (344 and 556 bp respectively). Interestingly, a number of *B. taylorii* and *B. grahamii* sequences originally obtained from bank voles were likely to have a mixed origin, as sequences were found to be identical to those from *B. taylorii* cluster E, which were exclusively found in wood mice. This sequence mosaicism was supported by at least three different algorithms in RDP3 and SplitsTree (*p*<0.05). Thus, Structure analysis disclosed evidence of recombination events among *B. taylorii* related genotypes, as well as between *B. taylorii* and *B. grahamii* genotypes, independent of the rodent species from which they were recovered.

**Figure 4:**
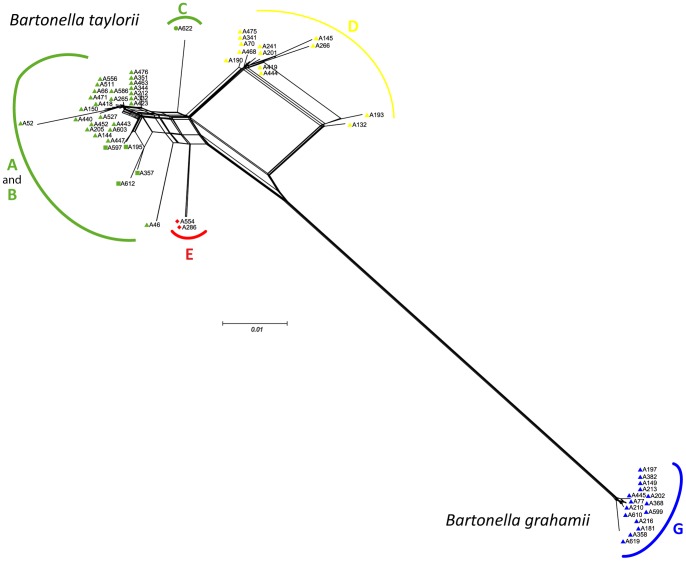
Neighbor-Net graph based on concatenated sequences of the six housekeeping genes of *Bartonella* genotypes. Note the bushy network structure among *Bartonella* strains indicative of pervasive homologous recombination.

These independent recombination events were further confirmed by performing a phylogenetic network analysis using the Neighbor-Net method. Evidence of extensive homologous recombination within the different genotypes, including cluster E, was supported by visual inspection of the bushy network structure with complex parallelogram formation (Figure 4).

### Diversity and allele sharing of virB5 coding sequences among *Bartonella* clusters

Due to the important role of the VirB/D4 type IV SS in host adaptability of *Bartonella* species [5], we investigated the diversity and homologous recombination events among the pilus component-coding *virB5* sequences.

*virB5* sequences analyse was performed using genotypes retrieved from bank voles and wood mice only. Overall, the coding sequences of virulence factor *virB5* showed 12 alleles undergoing a distinctive pattern of evolution. In relation to the entire *virB5* gene, calculation of the *dN*/*dS* ratio (1.145) disclosed a weak purifying selection and/or diversifying selection. Differences between *virB5* alleles were due to mutation, recombination events and the loss/gain of indels [46]. In all, the inferred VirB5 product ranged from 149 to 174 amino acids (aa) in length.

**Figure 5:**
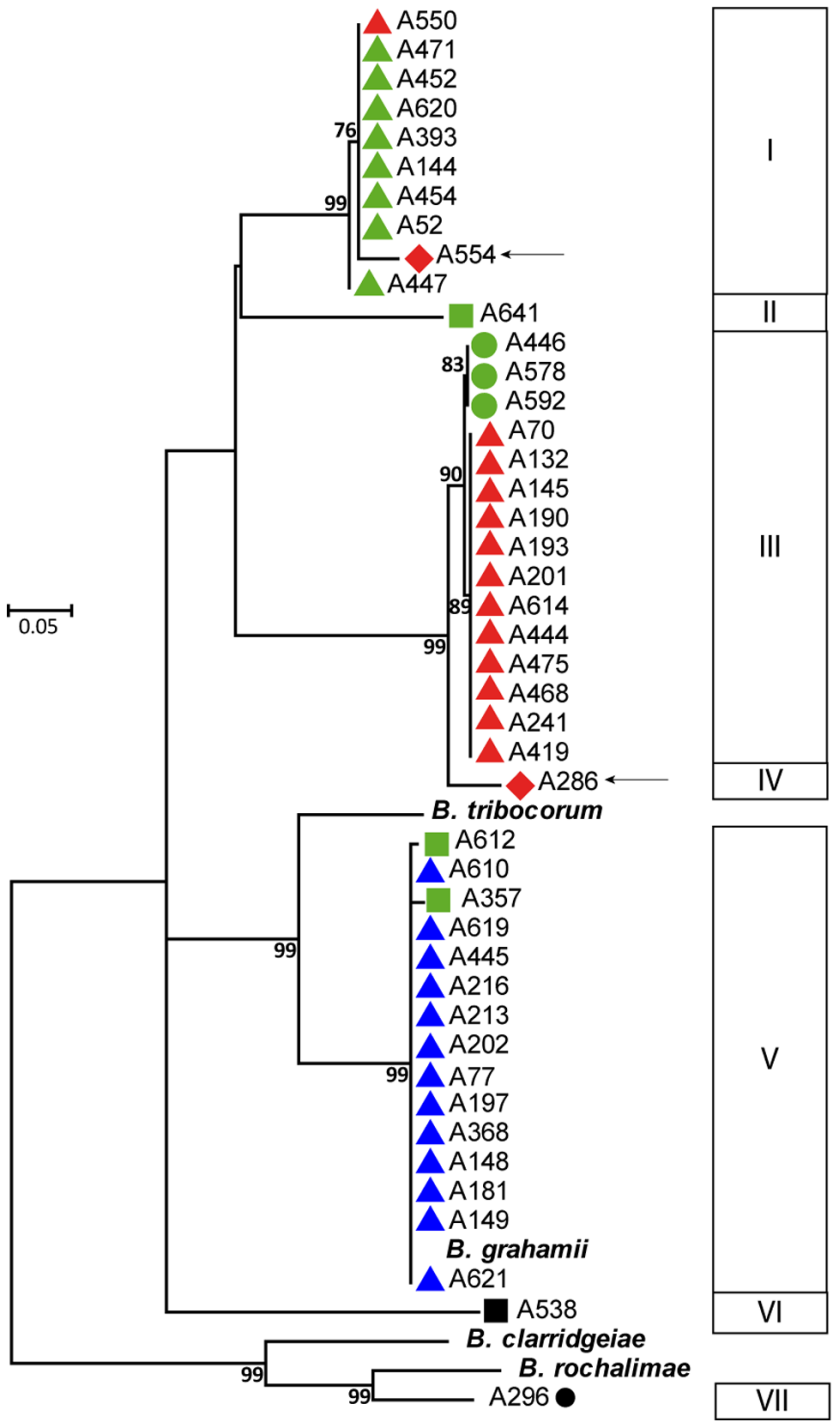
Phylogeny of *virB5* gene sequences using Maximum likelihood, with a GTR substitution model. Clusters in which *virB5* alleles were found are indicated by colors and shapes identical to Figure 1 (see legend). Bootstrap values higher than 80% are given at the nodes. Arrows indicate *virB5* sequences amplified and sequenced from strains obtained from wood mice.

A phylogenetic tree (Figure 5) was generated using maximum likelihood with a GTR substitution model. *virB5* sequences were clustered into seven clades (from clusters 1 to 7, in Figure 5). In contrast to housekeeping genes, phylogenetic analysis of *virB5* sequences merged those sequences related to *B. taylorii* cluster C (specific to bank voles) with that of *B. taylorii* cluster D (infecting mainly bank voles and to a lesser extent, field mice), with 1.1% nucleotide divergence (see cluster 3 in Figure 5). This phylogenetic analysis also uncovered a conspicuous pattern of homologous recombination of short intragenic regions and entire *virB5* sequences among the clusters, resulting in mosaic-like structures. The *virB5* sequence of genotype A550 (*B. taylorii*, cluster D with MLSA, recovered from bank voles) was identical to cluster A sequences (infecting mainly bank voles, and wood mice to a lesser extent). Likewise, the *virB5* sequence of genotype A554 (cluster E with MLSA, infecting wood mice) was merged with those from cluster A. Moreover, the *virB5* sequences of genotype A612 and A357 (cluster B infecting bank voles) were nearly identical to those of *B. grahamii* (exclusively infecting bank voles).

These results indicated that similar *virb5* sequences are distributed across the clades identified with MLSA analysis, clearly demonstrating that *virB5* alleles are not structured according to rodent host species.

## Discussion

By studying the diversity of *Bartonella* strains recovered from a rodent community comprising two main rodent species, we provide evidence of strong host-specific associations between *Bartonella* genotypes and their hosts. Interestingly, we also highlight the existence of many recombination events between different *Bartonella* species and genotypes, demonstrating that even though these genotypes do not share the same rodent host species, they should co-exist during their life cycle, probably within their arthropod vectors, providing opportunities for recombination.

Until now, the majority of studies describing *Bartonella* populations within rodent communities used a *gltA* gene sequence to identify and characterize *Bartonella* genotypes. As shown in this study, many recombination events occur within this gene. Thus, only using this gene might lead to biases resulting in the false identification of genotypes [28]. To obtain unbiased and more discriminatory *Bartonella* genotype identification, we used six housekeeping genes, as well as the host adaptability VirB5-encoding gene, enabling a more precise overview of *Bartonella* strain diversity from distinct rodent species. The present work unambiguously confirmed the high diversity of *Bartonella* genotypes recovered from woodland rodents as demonstrated in previous studies [16], [18], [47], with 63 different *Bartonella* genotypes retrieved from 195 infected animals among 550 captured. These genotypes were separated into three known *Bartonella* species: *B. taylorii*, *B. grahamii* and *B. doshiae*, commonly found in Europe, and one genotype related to *B. rochalimae-*like isolates obtained from wood mice in Sweden and Spain [15], [48]. The percentage of nucleotide divergence between this genotype and *B. rochalimae* is 8.2%, based on MLSA. As a nucleotide divergence greater than 5% within housekeeping genes is now considered as equivalent to the 70% DNA-DNA re-association criteria used for species demarcation, *B.rochalimae*-like strains isolated here and elsewhere in Europe thus correspond to a new species. Unfortunately, we failed to obtain a pure microbiological culture, therefore it will be named candidatus *Bartonella senartensis*, “Sénart” being the name of the forest from where the rodents were collected.

To highlight specific associations between *Bartonella* and their mammalian hosts, our strategy was to study the diversity of *Bartonella* strains isolated from different sympatric rodent species. Globally, the *B. taylorii* population, identified in 160 individuals (bank voles, field voles and wood mice), was much larger than the *B. grahamii* population, which was only recovered from 29 bank voles. *B. doshiae* and *B. rochalimae*-like sequences were the least abundant genotypes, recovered from six and one bank vole(s) respectively. Interestingly, we identified much higher diversity within the five *B. taylorii* clades (1.76% using MLSA), compared to *B. grahamii* (0.2%) which were grouped into a single cluster. These large differences in diversity were also corroborated by the *virB5* phylogeny, which distinguished nine different alleles for *B. taylorii*, while only one *virB5* sequence was identified for *B. grahamii*. Similar homogeneity within *B. grahamii* strains has been observed in both the UK and Sweden (0.1%) [26] while high polymorphism of this species has been observed in Asia [25]. Different hypothesis could explain this low diversity of *B. grahamii* in Europe. The first one is that *B. grahamii* originated in Asia and then spread recently in Europe by the introduction of its hosts. A second hypothesis might be that *B. grahamii* experienced in Europe a severe bottleneck (i.e., a reduction in population size) relatively recently, with too little time having elapsed for polymorphisms to re-accumulate. This bottleneck could be the consequence of a host shift from a sympatric rodent species not sampled in this study and bank voles. The low polymorphism rate within *B. grahamii* is also consistent with the simultaneous diversification of *B. taylorii* in multiple lineages, due to a possible rapid population expansion in order to occupy empty ecological niches following the proposed *B. grahamii* bottleneck.

Despite the small number of wood mice and field voles compared to bank voles, our study tends to demonstrate that the *Bartonella* population hosted by bank voles was more diverse than those retrieved from wood mice or field voles. Our results also strongly suggest that *B. taylorii* (clusters B-C), *B. grahamii*, *B. doshiae* and candidatus *Bartonella senartensis* might present host specificity for bank voles, and cluster E of *B. taylorii* for wood mice. A broader host range has been identified for some genotypes belonging to clusters A and D (of *B. taylorii*), that can infect both bank voles or wood mice, or bank voles and field voles respectively. Seasonal host density is known to drive which *Bartonella* species infects sympatric rodents [47]. However, in our study, vole and mouse concomitantly trapped on the same grids in April 2008 presented equivalent population abundances (Dr B. Pisanu, personal communication). Our results thus suggest that host density plays a minor role in explaining the differences in *Bartonella* infection between voles and mice. Rather, host susceptibility might have a major impact on the different patterns of *Bartonella* diversity according to rodent species. This hypothesis should be confirmed by a longitudinal analysis of *Bartonella* diversity in wood mice co-inhabiting with bank voles in Sénart.

Interestingly, the ratio of recombination to mutation (*r/m*) across the entire *Bartonella* population was estimated to be 4.06, which was much higher compared to that of *B. henselae* (0.1) [27]. In the particular case of *B. grahamii*, we obtained the high *r*/*m* ratio of 3.77, compared to 1.7 for *B. grahamii* strains published in the study by Berglund *et al*. [26]. This difference may be due to the population of *B. grahamii* isolates sampled (i.e., *B. grahamii* genotypes collected worldwide versus *B. grahamii* genotypes sampled from a small area in our study). Interestingly, even though *B. grahamii* strains had a low polymorphism rate, we did identify recombination events, suggesting that perhaps the resulting recombinants did not persist within bank voles, due to a possible inability to adapt to their host. Another finding was that the contribution of recombination in generating diversity among *B. taylorii* (*r*/*m*  = 6.81) was almost twice as high as that seen within *B. grahamii* (*r*/*m*  = 3.77). These results reflect the greater diversity of *B. taylorii* strains compared to *B. grahamii*, and are also consistent with the wider host range of *B. taylorii.*

Our analysis also demonstrated DNA exchanges between *Bartonella* genotypes infecting the same rodent species such as *B. taylorii* clusters A and D; *B. taylorii* cluster D and *B. grahamii* cluster G. Interestingly, recombination between wood mice-specific genotypes (cluster E) and bank vole-specific genotypes from *B. taylorii* and *B. grahamii* has also been highlighted, suggesting their co-occurrence during their life cycle, possible in a common arthropod vector. Interestingly, different rodent species in the Sénart forest had equivalent diversity of rodent flea communities, supporting the hypothesis of genetic exchange within the vector [49].

As for the mechanisms responsible for host specificity, our results clearly indicate that the *virB5* phylogeny is not sufficient to completely account for the pattern of rodent host association, and therefore must depend on additional more complex systems. In order to evaluate the potentially increased risk for humans to be infected with rodent-adapted *Bartonella* species due to possible host-shifting, understanding the molecular mechanisms of host specificity in areas where humans encounter rodents is extremely important. As suggested by recent genomic surveys, many putative host adaptability genes are essential for blood stream infection [5], [8] including other T4SS, adhesins, autotransporters. In this context, global analysis of all genomes is thus required in order to elucidate the subtle differences between closely related genotypes with different host ranges. Our study provides closely related genotypes harboring different host specificity patterns that should allow to further elucidate *Bartonella* host range preference at the molecular level by global genome comparison.

## Supporting Information

Figure S1 **Individual phylogenies of Bartonella genotypes constructed using internal sequences of six protein-coding genes (ftsZ, gltA, groEL, nuoG, ribC and rpoB).**
(PDF)

File S1 **This Includes Table S1 and Table S2.** Table S1. GenBank accession numbers for *ftsZ*, *gltA*, *groEL*, *ribC*, *rpoB* and *nuoG* sequences of *Bartonella* reference strains. Table S2. Accession numbers of sequences of the six protein-coding genes and the *virB5* gene from the 63 representative genotypes in GenBank/EMBL/DDBJ.
(DOC)
