# Supplementary figures and images for: Deciphering Bartonella Diversity, Recombination, and Host Specificity in a Rodent Community
Source: PLoS One. 2013 Jul 24;8(7):e68956. doi: 10.1371/journal.pone.0068956 (PMC3722228; doi:10.1371/journal.pone.0068956)

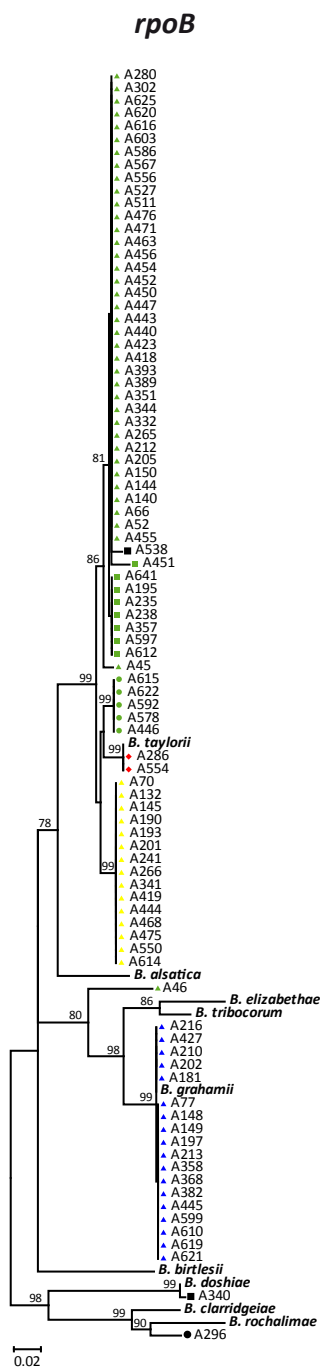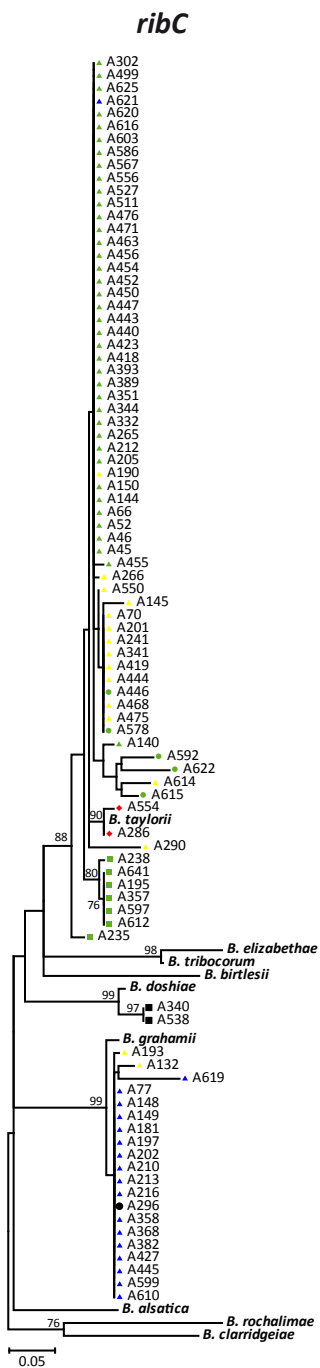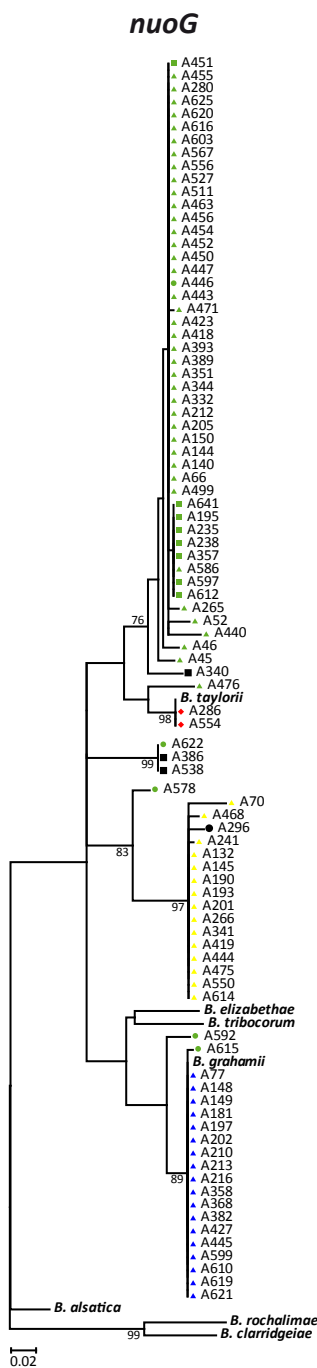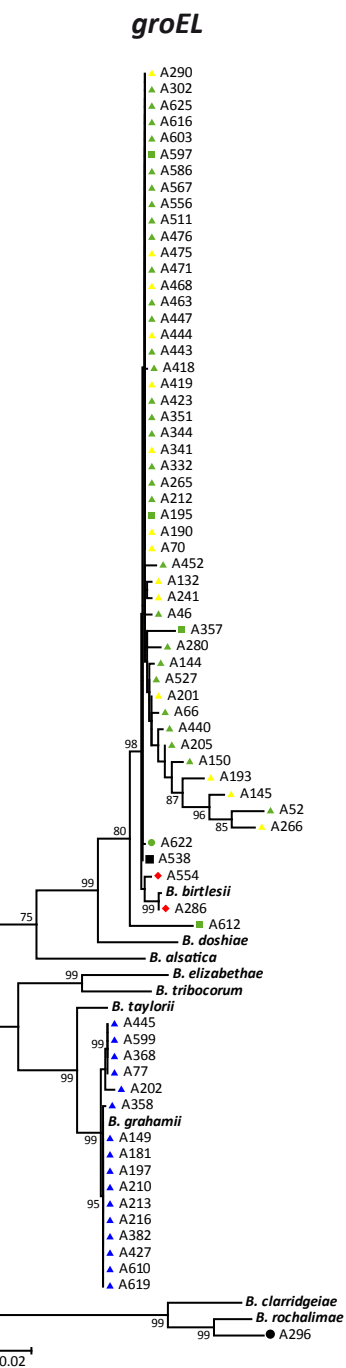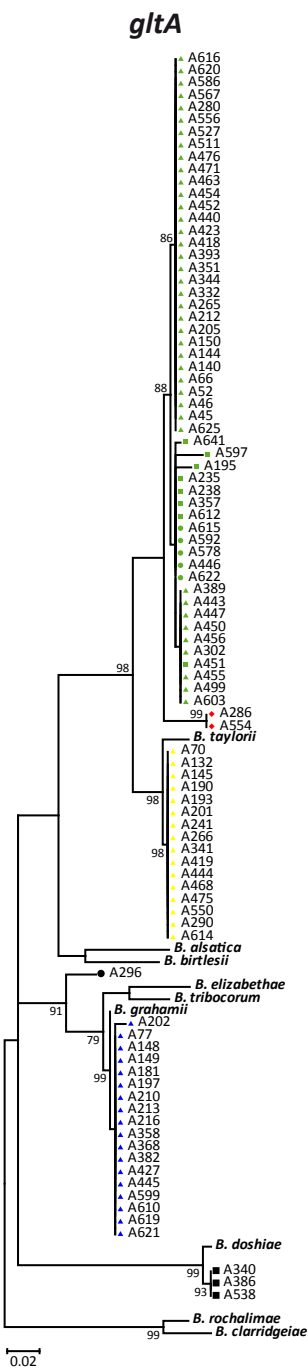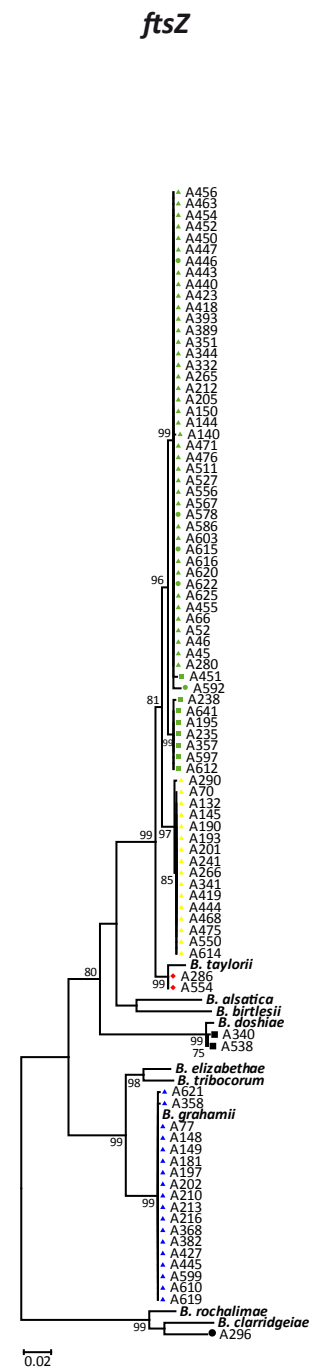

Supplement: Figure S1 — Individual phylogenies of Bartonella genotypes constructed using internal sequences of six protein-coding genes (ftsZ, gltA, groEL, nuoG, ribC and rpoB). (PDF) [file pone.0068956.s001.pdf]
