# Supplementary material for: Deciphering Bartonella Diversity, Recombination, and Host Specificity in a Rodent Community
Source: PLoS One. 2013 Jul 24;8(7):e68956. doi: 10.1371/journal.pone.0068956 (PMC3722228; doi:10.1371/journal.pone.0068956)
Supplement: File S1 — This Includes Table S1 and Table S2. Table S1. GenBank accession numbers for ftsZ, gltA, groEL, ribC, rpoB and nuoG sequences of Bartonella reference strains. Table S2. Accession numbers of sequences of the six protein-coding genes and the virB5 gene from the 63 representative genotypes in GenBank/EMBL/DDBJ. (DOC) [file pone.0068956.s002.doc]

**Supplementary Table S1. GenBank accession numbers for *ftsZ*, *gltA*, *groEL*, *ribC*, *rpoB* and *nuoG* sequences**

| **Species** | **Strain** | **Accession numbers** | | | | | |
| --- | --- | --- | --- | --- | --- | --- | --- |
|  |  | ***ftsZ*** | ***gltA*** | ***groEL*** | ***ribC*** | ***rpoB*** | ***nuoG*** |
| ***B. taylorii*** | M6 | AF467756 | Z70013 | AF304017 | AY116635 | AF165995 | EF659943 |
| ***B. doshiae*** | R18 | AF467754 | AF207827 | AF014832 | AY116627 | AF165991 | - |
| ***B. acomydis*** | KS2-1 | AB602545 | AB444979 | - | - | AB529942 | - |
| ***B. birtlesii*** | IBS 325 | AM690313 | AF204272 | AM690315 | AM690314 | AB196425 | - |
| ***B. callosciuri*** | BR1-1 | AB602541 | AB444977 | - | - | AB529929 | - |
| ***B. queenslandensis*** | AUST/NH12 | EU111776 | EU111798 | - | - | EU111787 | - |
| ***B. elizabethae*** | F9251 | AF467760 | Z70009 | AF014834 | AY116633 | AF165992 | EF659940 |
| ***B. tribocorum*** | CIP 105476 | NC_010161 | NC_010161 | NC_010161 | NC_010161 | NC_010161 | NC_010161 |
| ***B. rattimassiliensis*** | 15908 | AY515133 | AY515124 | AY515127 | AY515136 | AY515130 | - |
| ***B. grahamii*** | as4aup | NC_012846 | NC_012846 | NC_012846 | NC_012846 | NC_012846 | NC_012846 |
| ***B. phoceensis*** | 16120 | AY515135 | AY515126 | AY515129 | AY515138 | AY515132 | - |
| ***B. silvatica*** | Fuji 23-1 | AB440637 | AB242287 | AB440638 | AB440639 | AB242292 | - |
| ***B. alsatica*** | IBS 382 | AF467763 | AF204273 | AF299357 | AY116630 | AF165987 | EF659935 |
| ***B. vinsonii* subsp. *berkhoffii*** | 93-CO1 | AF467764 | BVU28075 | AF014836 | AF548031 | AF165989 | EF659937 |
| ***B. vinsonii* subsp. *vinsonii*** | Baker | AF467757 | Z70015 | AF014835 | AY116636 | AF165997 | EF659944 |
| ***B. vinsonii* subsp. *arupensis*** | OK94-513 | AF467758 | AF214557 | AF304016 | AY116631 | AY166582 | EF659936 |
| ***B. pachyuromydis*** | FN15-2 | AB602543 | AB444978 | - | - | AB602555 | - |
| ***B. jaculi*** | OY2-1 | AB602539 | AB444975 | - | - | AB529934 | - |
| ***B. washoensis*** | Sb944nv | AB292598 | AF470616 | AF484066 | AB292599 | AB292596 | EF659945 |
| ***B. henselae*** | Houston-1 | NC_005956 | NC_005956 | NC_005956 | NC_005956 | NC_005956 | NC_005956 |
| ***B. koehlerae*** | C-29 | AF467755 | AF176091 | AY116641 | AY116634 | AY166580 | EF659942 |
| ***B. quintana*** | Toulouse | NC_005955 | NC_005955 | NC_005955 | NC_005955 | NC_005955 | NC_005955 |
| ***B. rattaustraliani*** | AUST/NH14 | EU111774 | EU111796 | - | - | EU111785 | - |
| ***B. japonica*** | Fuji 18-1 | AB440633 | AB242289 | AB440634 | AB440635 | AB242288 | - |
| ***B. coopersplainsensis*** | AUST/NH20 | EU111781 | EU111803 | - | - | EU111792 | - |
| ***B. rochalimae*** | ATCC BAA-1498 | FN645461 | FN645459 | FN645466 | - | FN645459 | FN645459 |
| ***B. clarridgeiae*** | 73 | NC_014932 | NC_014932 | NC_014932 | NC_014932 | NC_014932 | NC_014932 |
| ***B. capreoli*** | IBS 193 | AB290192 | AF293392 | AB290190 | AB290194 | AB290188 | - |
| ***B. schoenbuchensis*** | R1 | AF467765 | AJ278183 | AY116642 | AY116628 | AY167409 | FN645509 |
| ***B. chomelii*** | A828 | AB290193 | AY254308 | AB290191 | AB290195 | AB290189 | - |
| ***B. bovis*** | 91-4 | - | AF293394 | - | AY116637 | AY166581 | EF659938 |
| ***B. bacilliformis*** | KC583 | NC_008783 | NC_008783 | NC_008783 | NC_008783 | NC_008783 | NC_008783 |
| ***B. tamiae*** | Th239 | DQ395178 | DQ395177 | DQ395179 | - | EF091855 | - |

**Supplementary Table S2. Accession numbers of sequences of the six protein-coding genes and the virB5 gene from the 63 representative genotypes in GenBank/EMBL/DDBJ**

| **Genotypes** | **ftsZ** | ***gltA*** | ***groEL*** | ***nuoG*** | ***ribC*** | ***rpoB*** | ***virB5*** |
| --- | --- | --- | --- | --- | --- | --- | --- |
| A132 | JX846096 | JX846160 | JX846221 | JX846268 | JX846330 | JX846393 | JX846478 |
| A140 | JX846097 | JX846161 |  | JX846269 | JX846331 | JX846394 | |
| A144 | JX846098 | JX846162 | JX846222 | JX846270 | JX846332 | JX846395 | JX846466 |
| A145 | JX846099 | JX846163 | JX846223 | JX846271 | JX846333 | JX846396 | JX846479 |
| A148 | JX846100 | JX846164 |  | JX846272 | JX846334 | JX846397 | JX846453 |
| A149 | JX846101 | JX846165 | JX846224 | JX846273 | JX846335 | JX846398 | JX846451 |
| A150 | JX846102 | JX846166 | JX846225 | JX846274 | JX846336 | JX846399 | |
| A181 | JX846103 | JX846167 | JX846226 | JX846275 | JX846337 | JX846400 | JX846452 |
| A190 | JX846104 | JX846168 | JX846227 | JX846276 | JX846338 | JX846401 | JX846480 |
| A193 | JX846105 | JX846169 | JX846228 | JX846277 | JX846339 | JX846402 | JX846481 |
| A195 | JX846106 | JX846170 | JX846229 | JX846278 | JX846340 | JX846403 | |
| A197 | JX846107 | JX846171 | JX846230 | JX846279 | JX846341 | JX846404 | JX846455 |
| A201 | JX846108 | JX846172 | JX846231 | JX846280 | JX846342 | JX846405 | JX846482 |
| A202 | JX846109 | JX846173 | JX846232 | JX846281 | JX846343 | JX846406 | JX846457 |
| A205 | JX846110 | JX846174 | JX846233 | JX846282 | JX846344 | JX846407 | |
| A213 | JX846111 | JX846175 | JX846234 | JX846283 | JX846345 | JX846408 | JX846458 |
| A216 | JX846112 | JX846176 | JX846235 | JX846284 | JX846346 | JX846409 | JX846459 |
| A235 | JX846113 | JX846177 |  | JX846285 | JX846347 | JX846410 | |
| A238 | JX846114 | JX846178 |  | JX846286 | JX846348 | JX846411 | |
| A241 | JX846115 | JX846179 | JX846236 | JX846287 | JX846349 | JX846412 | JX846489 |
| A265 | JX846116 | JX846180 | JX846237 | JX846288 | JX846350 | JX846413 | |
| A266 | JX846117 | JX846181 | JX846238 | JX846289 | JX846351 | JX846414 | |
| A286 | JX846118 | JX846182 | JX846239 | JX846290 | JX846352 | JX846415 | JX846490 |
| A296 | JX846119 | JX846183 | JX846240 | JX846291 | JX846353 | JX846416 | JX846493 |
| A340 | JX846150 | JX846214 |  |  | JX846384 | JX846447 | |
| A357 | JX846120 | JX846184 | JX846241 | JX846292 | JX846354 | JX846417 | JX846472 |
| A358 | JX846121 | JX846185 | JX846242 | JX846293 | JX846355 | JX846418 | |
| A368 | JX846122 | JX846186 | JX846243 | JX846294 | JX846356 | JX846419 | JX846454 |
| A393 | JX846123 | JX846187 |  | JX846295 | JX846357 | JX846420 | JX846467 |
| A419 | JX846124 | JX846188 | JX846244 | JX846296 | JX846358 | JX846421 | JX846488 |
| A440 | JX846125 | JX846189 | JX846245 | JX846297 | JX846359 | JX846422 | |
| A444 | JX846126 | JX846190 | JX846246 | JX846298 | JX846360 | JX846423 | JX846485 |
| A445 | JX846127 | JX846191 | JX846247 | JX846299 | JX846361 | JX846424 | JX846460 |
| A446 | JX846128 | JX846192 |  | JX846300 | JX846362 | JX846425 | JX846476 |
| A447 | JX846129 | JX846193 | JX846248 | JX846301 | JX846363 | JX846426 | JX846463 |
| A45 | JX846090 | JX846154 |  | JX846262 | JX846324 | JX846387 | |
| A452 | JX846130 | JX846194 | JX846249 | JX846302 | JX846364 | JX846427 | JX846469 |
| A454 | JX846131 | JX846195 |  | JX846303 | JX846365 | JX846428 | JX846465 |
| A46 | JX846091 | JX846155 | JX846216 | JX846263 | JX846325 | JX846388 | |
| A468 | JX846132 | JX846196 | JX846250 | JX846304 | JX846366 | JX846429 | JX846487 |
| A471 | JX846133 | JX846197 | JX846251 | JX846305 | JX846367 | JX846430 | JX846470 |
| A475 | JX846134 | JX846198 | JX846252 | JX846306 | JX846368 | JX846431 | JX846486 |
| A476 | JX846135 | JX846199 | JX846253 | JX846307 | JX846369 | JX846432 | |
| A52 | JX846092 | JX846156 | JX846217 | JX846264 | JX846326 | JX846389 | JX846464 |
| A527 | JX846136 | JX846200 | JX846254 | JX846308 | JX846370 | JX846433 | |
| A538 | JX846151 | JX846215 | JX846261 | JX846322 | JX846385 | JX846448 | JX846492 |
| A550 | JX846137 | JX846201 |  | JX846309 | JX846371 | JX846434 | JX846483 |
| A554 | JX846138 | JX846202 | JX846255 | JX846310 | JX846372 | JX846435 | JX846491 |
| A578 | JX846139 | JX846203 |  | JX846311 | JX846373 | JX846436 | JX846475 |
| A592 | JX846140 | JX846204 |  | JX846312 | JX846374 | JX846437 | JX846474 |
| A597 | JX846141 | JX846205 | JX846256 | JX846313 | JX846375 | JX846438 | |
| A610 | JX846142 | JX846206 | JX846257 | JX846314 | JX846376 | JX846439 | JX846462 |
| A612 | JX846143 | JX846207 | JX846258 | JX846315 | JX846377 | JX846440 | JX846471 |
| A614 | JX846144 | JX846208 |  | JX846316 | JX846378 | JX846441 | JX846484 |
| A615 | JX846145 | JX846209 |  | JX846317 | JX846379 | JX846442 | |
| A619 | JX846146 | JX846210 | JX846259 | JX846318 | JX846380 | JX846443 | JX846461 |
| A620 | JX846147 | JX846211 |  | JX846319 | JX846381 | JX846444 | JX846468 |
| A621 | JX846148 | JX846212 |  | JX846320 | JX846382 | JX846445 | JX846450 |
| A622 | JX846149 | JX846213 | JX846260 | JX846321 | JX846383 | JX846446 | |
| A641 | JX846152 | JX846153 |  | JX846323 | JX846386 | JX846449 | JX846473 |
| A66 | JX846093 | JX846157 | JX846218 | JX846265 | JX846327 | JX846390 | |
| A70 | JX846094 | JX846158 | JX846219 | JX846266 | JX846328 | JX846391 | JX846477 |
| A77 | JX846095 | JX846159 | JX846220 | JX846267 | JX846329 | JX846392 | JX846456 |

**Supplementary Figure S1 in file “Supp Mat FigS1”. Individual phylogenies of Bartonella genotypes constructed using internal sequences of six protein-coding genes (*ftsZ*, *gltA*, *groEL*, *nuoG*, *ribC* and rpoB).**

Colors and shapes are identical to Fig. 1. Genotypes with positions that are discordant with the concatenate-based phylogeny are indicated on each phylogeny; and are colored according to their position in the concatenated phylogeny. Bootstrap values higher than 75% are given at the nodes.
